# Clinical Decision‐Making and Guideline Alignment Among Brazilian Physical Therapists: Evidence From Standardized Carpal Tunnel Syndrome Vignettes

**DOI:** 10.1111/jep.70575

**Published:** 2026-08-31

**Authors:** Luciane Fernanda Rodrigues Martinho Fernandes, Brenda Thaís Alves Cardoso, Darlisson Bueno Paranhos, Vítor Hugo Mendes Castro, Amanda Avelar de Resende, Paula Regina Mendes da Silva Serrão

**Affiliations:** ^1^ Department of Applied Physiotherapy Universidade Federal do Triângulo Mineiro Uberaba Minas Gerais Brazil; ^2^ Graduate Program in Physical Education, Universidade Federal do Triângulo Mineiro Uberaba Minas Gerais Brazil; ^3^ Graduate Program in Physiotherapy Universidade Federal do Triângulo Mineiro and Universidade Federal de Uberlândia, Uberaba and Uberlândia Minas Gerais Brazil; ^4^ Department of Physiotherapy Universidade Federal de São Carlos, São Carlos São Paulo Brazil

**Keywords:** Carpal Tunnel Syndrome, clinical decision‐making, clinical practice guidelines, evidence‐based practice, guideline implementation, physical therapy

## Abstract

**Background:**

Clinical practice guidelines (CPGs) are intended to support evidence‐based clinical decision‐making. However, the extent to which physical therapists' clinical decisions align with guideline recommendations remains uncertain, particularly in Brazil.

**Objective:**

To evaluate the alignment of Brazilian physical therapists' clinical decisions with recommendations from CTS clinical practice guidelines and to examine whether general evidence‐based practice (EBP) knowledge, self‐reported guideline use, and condition‐specific guideline knowledge are associated with this alignment.

**Methods:**

A cross‐sectional study was conducted using an online questionnaire comprising two clinical vignettes representing mild and moderate CTS. Participants selected assessment and treatment strategies, which were scored using a weighted guideline‐alignment system based on the strength of the supporting evidence. Scores were expressed as percentages. Between‐group comparisons were performed using Welch's independent *t*‐tests, and effect sizes were estimated using Cohen's d.

**Results:**

A total of 282 physical therapists were included. Overall alignment with guideline recommendations was low (mean 46.2%, SD 10.7), with similarly low scores for assessment (45.9% ± 14.5) and intervention (46.5% ± 11.7). No significant associations were observed between guideline alignment and any of the investigated variables (all *p* > 0.05). Effect sizes were uniformly small. All participants selected at least one non‐recommended intervention, and 66.7% selected at least one non‐recommended assessment procedure.

**Conclusion:**

Clinical decisions made by Brazilian physical therapists showed limited alignment with CTS clinical practice guideline recommendations. General EBP knowledge, self‐reported guideline use, and condition‐specific guideline knowledge were not significantly associated with guideline alignment, and all observed effect sizes were small. These findings suggest that improving guideline implementation requires addressing factors beyond individual knowledge and highlight the importance of behavioural, organizational, and contextual determinants of clinical decision‐making.

## 1 Introduction

Evidence‐based clinical decision‐making is a primary objective of clinical practice guidelines (CPGs), which translate the best available evidence into recommendations intended to support clinical practice [1, 2]. However, publishing a guideline does not ensure that its recommendations will be incorporated into routine practice. Guideline implementation is a progressive process that involves awareness, agreement, adoption, and sustained adherence [3]. Its success depends not only on healthcare professionals but also on the characteristics of the guideline and the context in which care is delivered [4, 5]. Understanding how closely clinical decisions align with guideline recommendations is therefore essential for identifying gaps in guideline uptake and informing more effective implementation strategies [6].

Evaluating guideline alignment requires a clinical condition for which robust clinical practice guidelines provide explicit recommendations that can serve as an objective reference standard for clinical decision‐making. Among musculoskeletal disorders, carpal tunnel syndrome (CTS) provides an appropriate model for this purpose. It is the most common upper‐extremity entrapment neuropathy and results from compression of the median nerve within the carpal tunnel, leading to pain, paraesthesia, sensory impairment, and functional limitations [1, 2]. High‐quality CPGs offer explicit recommendations for the assessment and conservative management of CTS, based on systematic reviews of the available evidence [1, 2]. Together, these characteristics make CTS a suitable model for evaluating how closely clinical decisions align with guideline recommendations.

Studies across a range of musculoskeletal conditions have consistently shown that clinical decision‐making often deviates from guideline recommendations, with continued use of low‐value interventions and limited use of evidence‐supported practices [7, 8, 9]. A similar pattern has been reported for CTS, where studies conducted in different countries have described substantial variation in assessment and conservative management despite the availability of evidence‐based recommendations [10, 11, 12]. Taken together, these findings suggest that the availability of CPGs alone is insufficient to ensure their incorporation into routine clinical practice. This persistent evidence‐to‐practice gap has shifted attention toward understanding the determinants of guideline implementation.

Previous research has explored several dimensions of evidence‐based practice among physical therapists, including clinicians' knowledge of evidence‐based practice [4, 5], knowledge and use of clinical practice guidelines [6, 13], and self‐reported clinical behaviours [10, 11, 14]. Although this body of research has improved our understanding of guideline implementation, it relies largely on indirect measures and cannot determine whether clinicians' decisions are actually consistent with guideline recommendations. Clinical decisions represent the point at which evidence is translated into patient care through guideline recommendations. They therefore provide a more direct indicator of guideline uptake than intermediate implementation outcomes such as knowledge, attitudes, or self‐reported behaviours [3, 6]. This perspective shifts the focus from what clinicians know to the decisions they actually make in practice.

Whether these commonly investigated factors are sufficient to explain variation in guideline‐aligned clinical decision‐making remains unclear. Understanding where the evidence‐to‐practice pathway breaks down may help inform implementation strategies that target barriers beyond knowledge alone. Against this backdrop, this study aimed to evaluate the alignment of Brazilian physical therapists' clinical decisions with recommendations from clinical practice guidelines for carpal tunnel syndrome using standardized clinical case vignettes and to examine whether this alignment was associated with general evidence‐based practice (EBP) knowledge, self‐reported knowledge and use of clinical practice guidelines (CPGs), and condition‐specific CPG knowledge. We hypothesized that greater evidence‐based practice (EBP) knowledge, general knowledge of clinical practice guidelines (CPGs), CPG use, and CTS‐specific guideline knowledge would be associated with higher guideline‐alignment scores.

## 2 Methods

### 2.1 Study Design and Participants

This cross‐sectional study was conducted using an online survey. The target population comprised licensed physical therapists from all five geographic regions of Brazil [16]. Eligible participants had graduated from a Brazilian institution and had treated at least one patient with carpal tunnel syndrome (CTS) during their professional practice. Survey responses that did not meet the predefined data quality criteria were excluded from the analysis. Participants were recruited through social media, email, and messaging applications, resulting in a convenience sample.

### 2.2 Ethical Considerations

The study was approved by the Research Ethics Committee of the Federal University of Triângulo Mineiro (CAAE: 26412219.0.0000.5154). Electronic informed consent was obtained from all participants before access to the survey. The first page of the survey described the study objectives, procedures, estimated completion time, and provided the investigators' contact information. Participation was voluntary, and only participants who provided informed consent were allowed to proceed. Participant anonymity was ensured throughout the study. Data were collected and stored using Google Forms (Google LLC, Mountain View, CA, USA), with access restricted to the research team.

### 2.3 Survey Development

The online questionnaire was independently developed by two investigators (XX and YY) and administered using Google Forms. It was specifically designed for this study and was not adapted from an existing instrument. The clinical vignettes were developed from contemporary CTS clinical practice guidelines to represent scenarios commonly encountered in physical therapy practice. Reporting followed the Checklist for Reporting Results of Internet E‐Surveys (CHERRIES) [17].

Before data collection, the questionnaire underwent pilot testing with five physical therapist researchers to assess question order, content coverage, clarity, and technical feasibility of the online platform. Feedback obtained during pilot testing was incorporated into the final version.

The questionnaire consisted of two sections. The first collected participants' sociodemographic, educational, and professional characteristics, together with information on evidence‐based practice (EBP) knowledge and the knowledge and use of clinical practice guidelines. This study represents a secondary analysis of data collected through the same national survey previously used to describe participants' demographic and professional characteristics [16]. It addresses a different research question and evaluates different outcomes, specifically clinical decision‐making and guideline alignment.

The second section assessed clinical decision‐making using two standardized clinical vignettes, an approach that allows all participants to evaluate identical clinical scenarios and has been shown to provide a valid method for assessing physiotherapists' adherence to clinical practice guidelines through standardized clinical vignettes [15]. The vignettes represented patients with mild and moderate CTS. For each vignette, participants selected assessment and treatment strategies from pre‐specified options using four response categories (“Yes”, “Maybe”, “No”, and “I don't know”). The complete vignettes are available in Supporting Information S1.

### 2.4 Recruitment Process

The survey was distributed through a public web link, and all recruitment activities were conducted online. Invitations were disseminated through social media posts published by the research team, the Brazilian Association of Orthopaedic and Traumatology Physical Therapy and Rheumatology Physical Therapy (ABRAFITO), and the Regional Councils of Physical Therapy and Occupational Therapy (CREFITOs). In addition, the research team, ABRAFITO, and several CREFITOs distributed invitation emails through their mailing lists. Data were collected between May and September 2020.

### 2.5 Survey Administration

The survey was accessed through a web link. At the end of the questionnaire, participants could choose to receive a summary of the study findings by email.

Questions were presented in a fixed order, with no randomization. Adaptive questioning was used so that selected questions were displayed according to responses provided in previous sections.

All items required a response before participants could proceed to the next page. For each clinical vignette, participants were instructed to select “Yes” for at least three and no more than six items, assigning the remaining items to the other response categories. Responses could be reviewed and modified before final submission.

## 3 Response Rates

Because recruitment was open, the number of unique visitors who accessed the survey link could not be determined. Google Forms does not record users who access the link without starting the questionnaire; therefore, view and participation rates could not be calculated.

A total of 344 individuals responded to the electronic informed consent form. Two declined participation and were automatically directed to the end of the survey. Of the remaining 342 respondents, 60 were final‐year physical therapy students and were excluded because they did not meet the eligibility criteria. Following the exclusion of these 60 students, no additional participants were excluded based on the predefined response‐quality criterion, as all eligible participants selected “Yes” for at least three of the six assessment items in each vignette. The final sample consisted of 282 licensed physical therapists.

Because all survey items were mandatory, every submitted questionnaire was complete, and a separate completion rate could not be calculated. Google Forms records submission time but not survey start time; therefore, individual completion times could not be determined.

## 4 Preventing Multiple e−Ntries From the Same Individual

Access to the survey required authentication through a Google account, and only one response per account was permitted, preventing duplicate submissions. Cookies and IP address verification were not used because Google account authentication served as the primary mechanism for preventing multiple entries.

## 5 Classification of Clinical Practices and Development of the Guideline‐Alignment Score

All assessment and treatment options included in the clinical vignettes were classified according to the clinical practice guidelines for carpal tunnel syndrome (CTS) available at the time of data collection [18, 19]. The JOSPT clinical practice guideline [18] was used as the primary reference for item classification. The AAOS guideline [19] was consulted only for assessment or treatment items not addressed in the JOSPT guideline, following a predefined hierarchical approach. Each item was then assigned to one of four recommendation categories:

- **Recommended:** the guideline explicitly recommended the assessment or intervention based on sufficient supporting evidence (Grades A–C according to the guideline classification). Examples included nocturnal splinting, the Phalen test, the Boston Carpal Tunnel Questionnaire (BCTQ), and the Durkan compression test.
- **Not recommended:** the guideline explicitly recommended against the assessment or intervention because available evidence indicated a lack of benefit or potential harm. Examples included laser therapy, iontophoresis, therapeutic ultrasound, and neurodynamic testing.
- **Limited or conflicting evidence/no specific recommendation:** the guideline evaluated the assessment or intervention, but the available evidence was insufficient, inconsistent, or of low certainty to support a recommendation, or no recommendation was provided. Examples included neural mobilization, kinesio taping, and acupuncture.
- **Not CTS‐specific:** the assessment or intervention was unrelated to CTS and was included as a clinical distractor to evaluate the specificity of clinical decision‐making. Examples included Froment's sign, Adson's test, and the Finkelstein test. These items were excluded from score calculation.

Each item was independently classified by two investigators according to the predefined hierarchical approach and the direction and strength of the recommendations described in the clinical practice guidelines. Disagreements were resolved through consensus. The complete list of items, their classifications, and the rationale for each classification is provided in Supporting Information S2.

Because no published instrument was available to quantify the alignment of clinical decisions with CTS guideline recommendations, a guideline‐alignment score was specifically developed for this study. The score was based exclusively on the direction and strength of the recommendations contained in the guidelines available at the time of data collection.

Different weights were assigned according to the recommendation category. Recommended and not recommended items received a weight of 1.0, whereas items supported by limited or conflicting evidence received a weight of 0.5, reflecting the lower certainty of the underlying evidence.

For recommended items, responses of “Yes” received the full weight, “Maybe” received half the assigned weight, and both “No” and “I don't know” received zero points. For not recommended items, the scoring logic was reversed: “No” received the full weight, whereas “Yes”, “Maybe”, and “I don't know” received zero points. Items classified as having limited or conflicting evidence followed the same scoring approach as recommended items, but with proportionally reduced weights.

The overall alignment score was calculated as the ratio between the score obtained and the maximum possible score and expressed as a percentage, with higher values indicating greater alignment with guideline recommendations. The complete scoring system is presented in Table 1.

**Table 1:** Scoring system used to calculate guideline‐alignment scores.

Item classification	Weight	Yes	Maybe	No	I don't know
Recommended	1,0	1,0	0,5	0	0
Not recommended	1,0	0	0	1,0	0
Limited or conflicting evidence	0,5	0,5	0,25	0	0
Not CTS‐specific	—	—	—	—	—

*Note:* The overall alignment score was calculated as the ratio of the obtained score to the maximum possible score, expressed as a percentage. Higher scores indicate greater alignment with clinical practice guideline recommendations. Items classified as “Not CTS‐specific” were excluded from the score calculation.

For descriptive purposes, alignment scores were categorized as low (< 50%), moderate (50.0%–74.9%), or high (≥ 75%). These cut‐off values were defined a priori based on the midpoint of the scale (50%) and the 75% threshold adopted in previous studies of guideline adherence [12]. The categories were used exclusively for descriptive purposes and were not included in inferential analyses.

## 6 Outcomes

The primary outcome was the overall guideline‐alignment score, expressed as a percentage. Secondary outcomes included separate guideline‐alignment scores for assessment and treatment decisions, also expressed as percentages.

To further characterize clinical decision‐making, three binary variables were derived to identify whether participants included: (1) at least one assessment test not recommended by the guidelines; (2) at least one assessment test unrelated to CTS; or (3) at least one intervention not recommended by the guidelines. These secondary outcomes were intended to identify specific patterns of non‐guideline‐concordant clinical decision‐making that might not be captured by the overall alignment score.

## 7 Statistical Analysis

Responses were excluded if participants did not follow survey instructions to select “Yes” for at least three and no more than six items in each clinical vignette because this was considered evidence of inadequate questionnaire completion. Descriptive statistics were used to summarize participant characteristics and guideline‐alignment scores. Continuous variables were presented as means and standard deviations (SDs), whereas categorical variables were presented as absolute and relative frequencies. The distribution of alignment scores was assessed through visual inspection of histograms and quantile–quantile (Q–Q) plots, together with skewness and kurtosis statistics.

Alignment scores were compared between groups defined according to four predefined variables: (1) general evidence‐based practice (EBP) knowledge (yes/no), (2) knowledge of clinical practice guidelines (yes/no), (3) self‐reported use of clinical practice guidelines (yes/no), and (4) knowledge of CTS‐specific guidelines (yes/no). Given the sample size and the approximately symmetric distribution of alignment scores, between‐group comparisons were performed using Welch's independent‐samples *t*‐test, which is robust to unequal variances and unequal group sizes. Because the analyses were exploratory, no adjustment for multiple comparisons was applied. Statistical significance was set at *p* < 0.05.

For descriptive purposes, the proportion of guideline‐aligned responses was also calculated for each assessment and treatment option individually and is presented graphically.

All statistical analyses were performed using IBM SPSS Statistics for Windows, Version 28.0 (IBM Corp., Armonk, NY, USA). Independent‐samples *t* tests were used to compare mean guideline‐alignment scores between groups. Cohen's *d* effect sizes were calculated separately in Microsoft Excel (Microsoft Corp., Redmond, WA, USA), as the difference between group means divided by the pooled standard deviation, using the means, standard deviations, and sample sizes obtained from the SPSS output. Effect sizes were interpreted as trivial (< 0.20), small (0.20–0.49), moderate (0.50–0.79), or large (≥ 0.80) [20].

## 8 Results

A total of 282 physical therapists were included in the analysis. Most participants were women (67.7%) and aged 26–35 years (51.4%). Overall, 53.5% held a specialist qualification, and 25.9% had completed a master's or doctoral degree. More than half (56.4%) reported previous experience in hand rehabilitation. Regarding evidence‐based practice (EBP) and clinical practice guidelines (CPGs), 97.2% reported knowledge of EBP, 84.0% reported general knowledge of CPGs, and 66.7% reported using CPGs in clinical practice. In contrast, only 20.2% reported specific knowledge of CTS guidelines (Table 2).

**Table 2:** Participant characteristics (*n *= 282).

Variable	Category	*n* (%)
Sociodemographic characteristics		
Sex	Female	191 (67.7)
	Male	91 (32.3)
Age group (years)	18–25 years	41 (14.5)
	26–35 years	145 (51.4)
	36–45 years	75 (26.6)
	Over 46 years	21 (7.4)
Educational and professional characteristics		
Highest academic qualification	Bachelor's degree	58 (20.6)
	Specialist degree	151 (53.5)
	Master's or doctoral degree	73 (25.9)
Years of clinical experience	up to 5 years	132 (46.8)
	6–10 years	54 (19.1)
	11–20 years	77 (27.3)
	above 20 years	19 (6.7)
Experience in hand rehabilitation	Yes	159 (56.4)
	No	123 (43.6)
Knowledge and use of EBP and clinical practice guidelines		
Self‐reported application of evidence‐based practice^a^	EBP is essential for clinical decision‐making	190 (67.4)
	I use EBP occasionally	78 (27.7)
	I do not use EBP	14 (5.0)
Self‐reported EBP knowledge	Yes	274 (97.2)
	No	8 (2.8)
General guideline knowledge	Yes	237 (84.0)
	No	45 (16.0)
Guideline use	Yes	188 (66.7)
	No	94 (33.3)
CTS‐specific guideline knowledge	Yes	57 (20.2)
	No	225 (79.8)

Abbreviations: CTS, carpal tunnel syndrome; EBP, evidence‐based practice.

^a^ Assessed using a single self‐reported item with three mutually exclusive response options; reflects participants' perceived attitude rather than observed clinical behavior.

**Figure 1:**
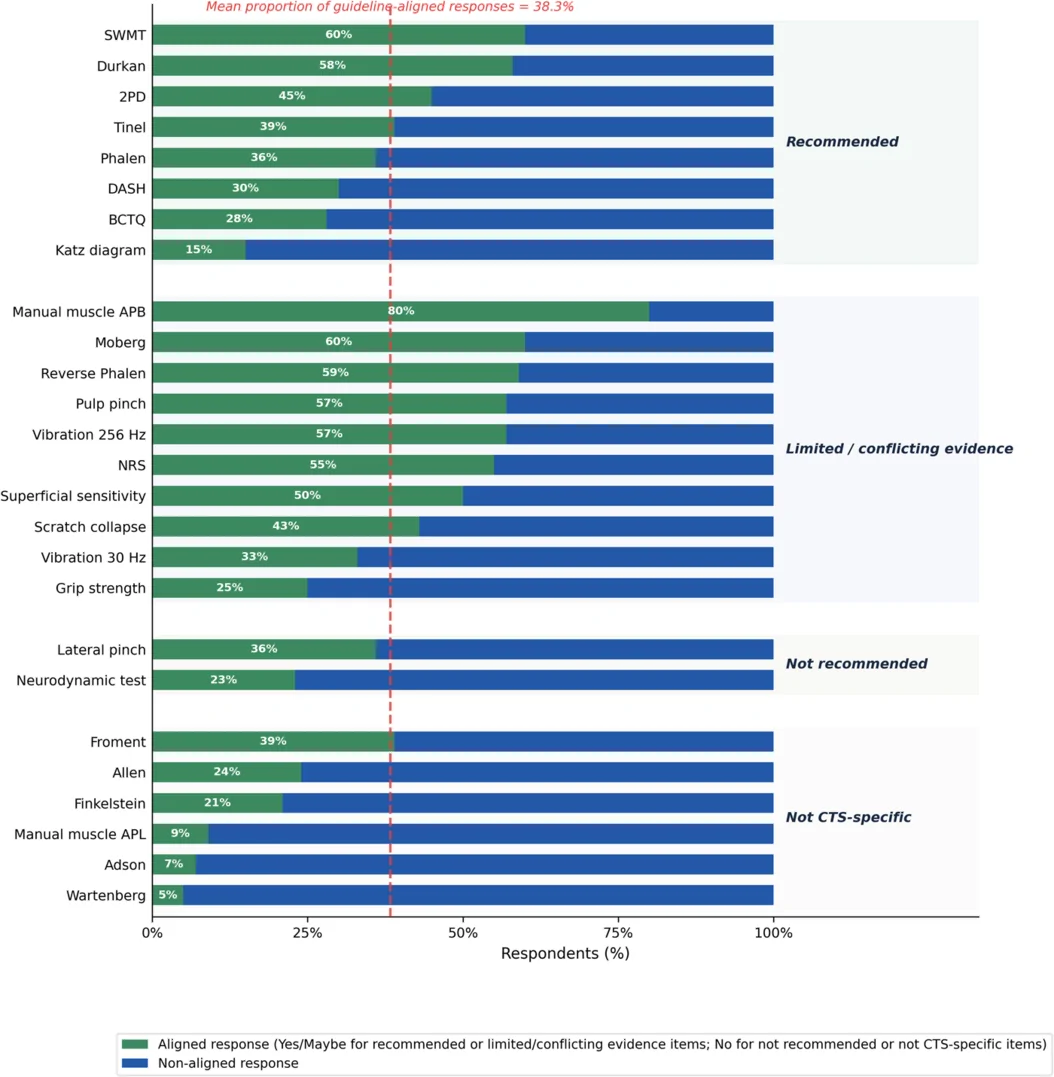
Proportion of guideline‐aligned responses for individual assessment options. *Note:* The dashed line represents the unweighted mean proportion of guideline‐aligned responses across all assessment items. It should not be interpreted as the participant‐level weighted guideline‐alignment score reported in Table 3.

Figure 1 illustrates the proportion of guideline‐aligned responses for individual assessment options included in the clinical vignettes. Among recommended assessment strategies, the Semmes–Weinstein monofilament test (60%) and the Durkan compression test (58%) showed the highest proportions of aligned responses, whereas the Katz hand diagram (15%), the Boston Carpal Tunnel Questionnaire (28%), and the DASH questionnaire (30%) showed the lowest. Several assessment procedures supported by limited or conflicting evidence were frequently selected, and 23% of participants selected the neurodynamic test despite its classification as not recommended.

**Figure 2:**
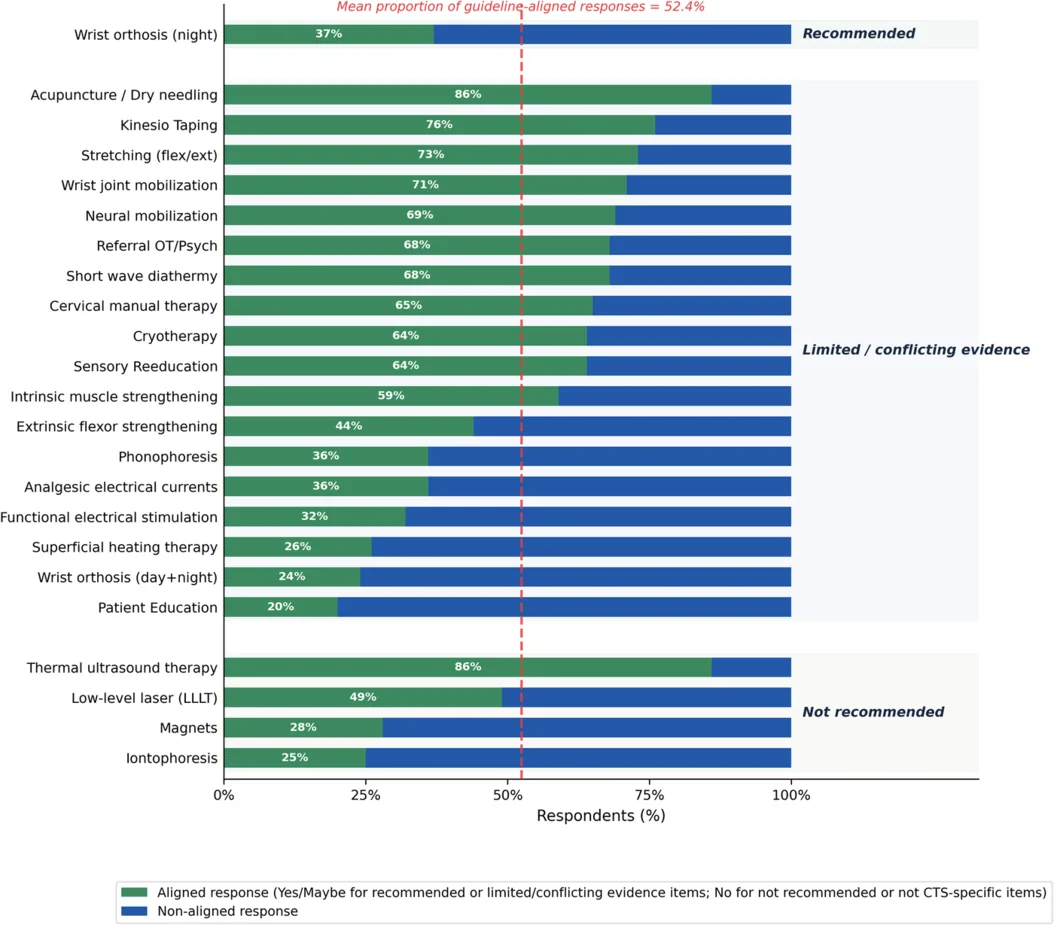
Proportion of guideline‐aligned responses for individual treatment options. *Note: T*he dashed line represents the unweighted mean proportion of guideline‐aligned responses across all treatment items. It should not be interpreted as the participant‐level weighted guideline‐alignment score reported in Table 3.

Figure 2 illustrates the proportion of guideline‐aligned responses for individual treatment options. Among treatment options, night wrist orthosis—the only recommended intervention—was selected by only 37% of participants. Conversely, several interventions supported by limited or conflicting evidence, including acupuncture (86%), kinesio taping (76%), stretching (73%), and wrist joint mobilization (71%), were frequently selected. Thermal ultrasound therapy, a non‐recommended intervention, was selected by 86% of participants.

The overall guideline‐alignment score was low (mean 46.2%, SD 10.7; 95% CI 44.93–47.43), indicating that participants' clinical decisions aligned with fewer than half of the evaluated guideline recommendations. Similar findings were observed for both assessment (45.9%, SD 14.5) and treatment decisions (46.5%, SD 11.7) (Table 3).

**Table 3:** Overall and domain‐specific guideline‐alignment scores (*n* = 282).

Outcome	Mean (SD)	95% CI
Assessment alignment score (%)	45.9 ± 14.5	44.20–47.60
Treatment alignment score (%)	46.5 ± 11.7	45.11–47.86
Overall alignment score (%)	46.2 ± 10.7	44.93–47.43

*Note:* Scores were calculated using a weighted scoring system based on the strength of evidence of each recommendation. Guideline‐alignment scores are expressed as percentages, with higher scores indicating greater alignment between clinical decisions and recommendations from clinical practice guidelines.

Abbreviations: CI, confidence interval; SD, standard deviation.

Inspection of individual assessment and treatment items showed that alignment was particularly low for several recommended assessment procedures and for the only recommended intervention (night wrist orthosis), whereas several interventions supported by limited or conflicting evidence—and even interventions explicitly discouraged by the guidelines—were frequently selected. This pattern suggests that low alignment was not driven by isolated decisions but reflected a broad tendency to diverge from guideline recommendations.

None of the investigated variables was significantly associated with the overall guideline‐alignment score (Table 4). Mean scores were similar regardless of participants' reported knowledge of EBP (46.22% vs. 44.83%; *p* = 0.652), general knowledge of CPGs (45.72% vs. 48.62%; *p* = 0.105), or self‐reported use of CPGs (46.18% vs. 46.18%; *p* = 0.997). Participants who reported specific knowledge of CTS guidelines achieved a numerically higher alignment score than those without such knowledge (48.71% vs. 45.54%; mean difference 3.17 percentage points; 95% CI − 0.46 to 6.81; *p* = 0.086; Cohen's *d* = 0.30), although the difference was not statistically significant.

**Table 4:** Overall guideline‐alignment scores according to participants' evidence‐based practice and guideline‐related characteristics (*n* = 282).

Variable	Group	Mean (SD)	[95% CI]	Mean difference (95% CI)	*p*	Cohen's *d*	Magnitude
Knowledge of evidence‐based practice	Yes (*n* = 274)	46.22 (10.7)	[44.94–47.50]	1.39 (−5.63 to 8.41)	0.652^a^	0.13	Negligible
	No (*n* = 8)^b^	44.83 (8.19)	[37.98–51.68]				
Knowledge of clinical practice guidelines	Yes (*n* = 237)	45.72 (10.6)	[44.37– 47.07]	−2.90 (−6.43 to 0.63)	0.105^a^	0.27	Small
	No (*n* = 45)	48.62 (10.9)	[45.34–51.90]				
Self‐reported use of clinical practice guidelines	Yes (*n* = 188)	46.18 (10.9)	[44.60–47.80]	0.00 (−2.60 to 2.61)	0.997^a^	0.00	Negligible
	No (*n* = 94)	46.18 (10.2)	[44.09–48.27]				
Knowledge of CTS clinical practice guidelines	Yes (*n* = 57)	48.71 (12.8)	[45.31–52.11]	3.17 (−0.46 to 6.81)	0.086^a^	0.30	Small
	No (*n* = 225)	45.54 (9.98)	[44.22–46.85]				

*Note:* Mean differences were calculated as the difference between the “Yes” and “No” groups. Between‐group comparisons were performed using Welch's independent‐samples *t*‐test. Effect sizes (Cohen's *d*) were interpreted as trivial (< 0.20), small (0.20–0.49), moderate (0.50–0.79), or large (≥ 0.80). Positive values indicate higher guideline‐alignment scores in the “Yes” group. Variables were coded according to participants' self‐reported responses (“Yes”/“No”).

Abbreviations: CI confidence interval; CTS, carpal tunnel syndrome; SD, standard deviation.

a Welch's independent‐samples *t*‐test.

b The “No” subgroup for evidence‐based practice knowledge included only eight participants; therefore, these results should be interpreted with caution.

The selection of practices not supported by guideline recommendations was common across all domains. Two‐thirds of participants (66.7%) selected at least one assessment test that was not recommended by the guidelines, and 85.1% selected at least one assessment test unrelated to CTS. All participants (100%) selected at least one intervention that was not recommended by the guidelines (Table 5).

**Table 5:** Proportion of participants selecting at least one non‐recommended or non‐CTS‐specific assessment test or intervention (*n* = 282).

Variable	*n* (%)
Use of non‐recommended assessment tests	188 (66.7)
Use of non‐CTS‐specific tests	240 (85.1)
Use of non‐recommended interventions	282 (100.0)

*Note:* Non‐recommended and non‐CTS‐specific practices were classified according to the recommendations of Erickson et al. (2019) and Graham et al. (2016).

Participants could select more than one item within each category.

## 9 Discussion

The main finding of this study was that Brazilian physical therapists showed limited alignment between their clinical decisions and clinical practice guideline recommendations for carpal tunnel syndrome (CTS), both for assessment and intervention decisions. Despite the high proportion of participants reporting knowledge of evidence‐based practice (EBP), awareness of clinical practice guidelines (CPGs), and self‐reported guideline use, participants' decisions aligned with fewer than half of the recommendations evaluated. Together, these findings reveal a substantial gap between the availability of evidence‐based recommendations and their incorporation into clinical decision‐making, highlighting that the existence of CPGs alone does not ensure their implementation in clinical practice.

This pattern is consistent with previous studies across a range of musculoskeletal conditions showing that clinical decision‐making frequently departs from guideline recommendations, with continued use of low‐value interventions and underutilization of evidence‐based care [7, 8, 9]. Similar findings have been reported for CTS in the United States, Italy, and Australia, where substantial variation in assessment and conservative management persists despite the availability of evidence‐based clinical practice guidelines [10, 11, 12]. Together, these studies indicate that the availability of high‐quality guidelines alone is insufficient to ensure their implementation in routine practice. Our findings build on this evidence by showing that the same implementation gap exists among Brazilian physical therapists and by directly quantifying the extent to which clinical decisions align with guideline recommendations using standardized clinical vignettes.

None of the variables investigated was significantly associated with guideline alignment. Although most participants reported being familiar with evidence‐based practice and clinical practice guidelines, these attributes were not reflected in more guideline‐concordant clinical decisions. This finding is consistent with previous research involving Brazilian physical therapists, which has shown widespread recognition of the importance of evidence‐based practice alongside limited use of clinical practice guidelines and primary evidence sources in everyday clinical decision‐making [13]. Taken together, these findings suggest that knowledge of, and familiarity with, clinical practice guidelines are important prerequisites for evidence‐based practice but are unlikely to be sufficient, on their own, to ensure their consistent translation into clinical decisions.

An unexpected finding was that participants who reported no general knowledge of clinical practice guidelines achieved a numerically higher guideline‐alignment score than those reporting such knowledge, although this difference was not statistically significant and should therefore be interpreted with caution. The relatively small number of participants reporting no general guideline knowledge (*n* = 45), compared with those reporting such knowledge (*n* = 237), may have reduced the precision of this comparison. In addition, because general guideline knowledge was assessed by self‐report, perceived familiarity with clinical practice guidelines may not accurately reflect actual knowledge or its application in clinical decision‐making. Although participants reporting specific knowledge of CTS clinical practice guidelines also showed numerically higher guideline‐alignment scores, this difference was likewise not statistically significant. Together, these findings reinforce that knowledge alone may be insufficient to explain guideline‐concordant clinical decisions, which are likely influenced by multiple individual and contextual factors.

This interpretation is consistent with both classical and contemporary implementation frameworks. The model proposed by Pathman et al. conceptualizes guideline implementation as a sequential process progressing from awareness and agreement to adoption and sustained adherence, indicating that knowledge represents only the first step toward implementation. Contemporary implementation research further emphasizes that successful guideline uptake depends on interactions between individual, guideline‐related, and organizational factors [4, 5, 6]. From this perspective, the absence of an association between knowledge‐related variables and guideline alignment suggests that the principal barriers may arise during the translation of knowledge into clinical decisions rather than during knowledge acquisition itself.

Participants who reported specific knowledge of CTS clinical practice guidelines achieved numerically higher guideline‐alignment scores than those without such knowledge; however, this difference did not reach statistical significance. The relatively small number of participants reporting specific guideline knowledge may have reduced the precision of the estimates and limited statistical power to detect small between‐group differences. Although the observed effect size (Cohen's *d* = 0.30) is compatible with a small association, the corresponding confidence interval included the null value, and therefore, this finding should be interpreted cautiously. Consequently, our results neither support nor exclude a potential contribution of condition‐specific guideline knowledge to guideline‐concordant clinical decision‐making.

The guideline‐alignment score developed for this study should be interpreted as a measure of agreement between clinical decisions and explicit guideline recommendations rather than as a measure of professional competence, quality of care, or clinical performance. Its purpose was to quantify the extent to which clinical decisions made in response to standardized clinical scenarios were consistent with evidence‐based recommendations for CTS. As such, the score provides an indicator of guideline implementation in clinical decision‐making, rather than an overall assessment of clinicians' professional performance.

These findings have important implications for both implementation research and clinical practice. Strategies focused solely on increasing clinicians' knowledge of evidence‐based practice or improving awareness of clinical practice guidelines are unlikely to produce meaningful changes in clinical decision‐making when implemented in isolation. Instead, future implementation strategies should adopt multifaceted approaches that simultaneously address individual, organizational, and contextual barriers to guideline uptake. From an implementation science perspective, clinical decisions may represent a more proximal implementation outcome than knowledge, attitudes, or self‐reported behaviors because they more directly reflect whether guideline recommendations are translated into patient care. Consequently, assessing clinical decision‐making may provide a more informative approach for evaluating the implementation of clinical practice guidelines than relying exclusively on intermediate implementation outcomes.

This study has several strengths. To our knowledge, it is the first study conducted in Brazil to directly quantify the alignment between physical therapists' clinical decisions and recommendations from CTS clinical practice guidelines using standardized clinical vignettes. Participants were recruited from all five geographical regions of the country, increasing the geographical diversity of the sample. In addition, unlike previous studies that assessed guideline adherence using categorical classifications, we developed a continuous guideline‐alignment score weighted according to the strength of the underlying recommendations, providing a more sensitive measure of the extent to which clinical decisions reflected current evidence.

Some limitations should be acknowledged. Convenience sampling may have preferentially attracted physical therapists with greater interest in evidence‐based practice, potentially overestimating knowledge of EBP and familiarity with clinical practice guidelines compared with the broader Brazilian physical therapist population. Although clinical vignettes are widely used to investigate clinical decision‐making under standardized conditions and reduce variability inherent to real clinical encounters, they cannot fully capture the complexity of routine practice, including patient interaction, resource availability, and organizational influences. In addition, the cross‐sectional design precludes causal inference and limits the findings to observed associations. Finally, the clinical practice guidelines used as the reference standard corresponded to the versions available at the time of data collection (2020). Although updated versions were published during manuscript preparation [1, 2], the recommendations most relevant to the conservative management of CTS remained largely unchanged.

Taken together, our findings suggest that low alignment between clinical decisions and guideline recommendations reflects a challenge that extends beyond individual knowledge. Closing the evidence‐to‐practice gap is likely to require implementation strategies that address the behavioral, organizational, and contextual determinants of guideline uptake. Future research should focus on identifying these determinants more precisely and on evaluating implementation interventions designed to improve guideline uptake and its translation into clinical decision‐making.

## 10 Conclusion

Brazilian physical therapists showed limited alignment between their clinical decisions and recommendations from clinical practice guidelines for carpal tunnel syndrome across both assessment and treatment decisions. Neither evidence‐based practice knowledge, awareness and self‐reported use of clinical practice guidelines, nor condition‐specific guideline knowledge was significantly associated with guideline alignment. These findings suggest that improving guideline‐concordant clinical decision‐making will require implementation strategies that address behavioural, organizational, and contextual determinants of guideline uptake rather than focusing exclusively on increasing clinicians' knowledge.

## Author Contributions

**Luciane Fernanda Rodrigues Martinho Fernandes:** conceptualization, methodology, formal analysis, supervision, funding acquisition, writing – review and editing. **Brenda Thaís Alves Cardoso:** data curation, writing – review and editing. **Darlisson Bueno Paranhos:** data curation, writing – review and editing. **Vítor Hugo Mendes Castro:** data curation, writing – review and editing. **Amanda Avelar de Resende:** investigation, data curation, writing – original draft. **Paula Regina Mendes da Silva Serrão:** conceptualization, methodology, writing – review and editing.

## Ethics Statement

The study was approved by the Research Ethics Committee of the Federal University of Triângulo Mineiro (CAAE: 26412219.0.0000.5154).

## Conflicts of Interest

The authors declare no conflicts of interest.

## Declaration

During the preparation of this manuscript, the authors used ChatGPT (OpenAI) to assist with language editing, improve clarity and readability, and refine the presentation of the text. Claude (Anthropic) was used to assist in the design and graphical presentation of Figures 1 and 2 and the Graphical Abstract, based exclusively on data, analyses, and scientific content produced by the authors. All AI‐assisted outputs were critically reviewed, revised, and approved by the authors, who take full responsibility for the accuracy, interpretation, and integrity of the manuscript.

## Supporting information


Supporting File 1

Supporting File 2

Supporting File 3

## Data Availability

The data that support the findings of this study are available from the corresponding author upon reasonable request.
