# Supplementary material for: Clinical Decision‐Making and Guideline Alignment Among Brazilian Physical Therapists: Evidence From Standardized Carpal Tunnel Syndrome Vignettes
Source: J Eval Clin Pract. 2026 Aug 31;32(6):e70575. doi: 10.1111/jep.70575 (PMC13527589; doi:10.1111/jep.70575)
Supplement: Supplementary file 1 [file JEP-32-0-s003.pdf]

Supplementary Table S2. Classification of assessment and treatment items according to clinical practice guidelines for carpal tunnel syndrome.

Domain	Instrument	Raw Variable	Category	Guideline	Level of Evidence	Guideline Classification	Justification	Principal score
Assessment	Manual muscle APB	AxAPBSR_raw	Strength	JOSPT	D	Limited / Conflicting	Conflicting evidence	No
Assessment	2PD	Ax2PD_raw	Sensory	JOSPT	A	Recommended	Recommended for sensory assessment	Yes
Assessment	Superficial sensitivity	AxSS_raw	Sensory	JOSPT	C	Limited / Conflicting	Limited evidence	No
Assessment	SWMT	AxSWMT_raw	Sensory	JOSPT	A	Recommended	Standard sensory test	Yes
Assessment	Tinel	AxTinel_raw	Special tests	JOSPT	B	Recommended	Part of diagnostic cluster	Yes
Assessment	Grip strength	AxGripSR_raw	Strength	JOSPT	C	Limited / Conflicting	Useful for baseline assessment but limited for follow-up	No
Assessment	Phalen	AxPhalen_raw	Special tests	JOSPT	B	Recommended	Recommended provocative test	Yes
Assessment	Katz diagram	AxKatz_raw	Questionnaire	JOSPT	B	Recommended	Validated diagnostic tool	Yes
Assessment	BCTQ	AxBCTQ_raw	Questionnaire	JOSPT	B	Recommended	Recommended for symptom and function assessment	Yes
Assessment	Lateral pinch	AxLPSR_raw	Strength	JOSPT	A	Not recommended	Not recommended as outcome measure	Yes
Assessment	Vibration 256 Hz	AxTF256_raw	Sensory	JOSPT	D	Limited / Conflicting	Conflicting evidence	No
Assessment	Durkan	AxDurkan_raw	Special tests	JOSPT	B	Recommended	Recommended compression test	Yes
Assessment	Vibration 30 Hz	AxTF30_raw	Sensory	JOSPT	D	Limited / Conflicting	Conflicting evidence	No
Assessment	Pulp pinch	AxPPSR_raw	Strength	JOSPT	C	Limited / Conflicting	Permitted use with limited evidence	No
Assessment	Reverse Phalen	AxRPhalen_raw	Special tests	-	-	Limited / Conflicting	Not addressed in CTS clinical practice guidelines	No
Assessment	DASH	AxDASH_raw	Questionnaire	JOSPT	B	Recommended	Recommended for functional assessment	Yes
Assessment	Neurodynamic test	AxNDT_raw	Special tests	AAOS	Moderate	Not recommended	Not recommended for diagnosis	Yes
Assessment	Froment	AxFroment_raw	Special tests	-	-	Not CTS-specific	Assesses ulnar nerve function	No
Assessment	Scratch collapse	AxSCT_raw	Special tests	JOSPT	D	Limited / Conflicting	Conflicting evidence	No
Assessment	Wartenberg	AxWatenberg_raw	Special tests	-	-	Not CTS-specific	Associated with ulnar nerve dysfunction	No
Assessment	Adson	AxAdson_raw	Special tests	-	-	Not CTS-specific	Assesses thoracic outlet syndrome	No
Assessment	Finkelstein	AxFinkelstein_raw	Special tests	-	-	Not CTS-specific	Assesses De Quervain tenosynovitis	No
Assessment	Allen	AxAllen_raw	Special tests	-	-	Not CTS-specific	Assesses vascular integrity	No
Assessment	Moberg	AxDellon_raw	Functional	JOSPT	C	Limited / Conflicting	Dexterity assessment supported by limited evidence	No
Assessment	Manual muscle APL	AxAPLSR_raw	Strength	-	-	Not CTS-specific	Not related to CTS	No
Assessment	NRS	AxNRS_raw	Questionnaire	-	-	Limited / Conflicting	Non-specific measure of pain intensity	No
Intervention	Patient Education	INT_PtEduc_raw	Education	JOSPT	C	Limited / Conflicting	Limited to ergonomic education regarding mouse and keyboard use.	No
Intervention	Thermal ultrasound therapy	INT_US_raw	Physical agents	JOSPT	C	Not recommended	Explicitly not recommended	Yes
Intervention	Cryotherapy	INT_Cryo_raw	Physical agents	-	-	Limited / Conflicting	Not addressed in CTS clinical practice guidelines	No
Intervention	Magnets	INT_Magnet_raw	Physical agents	JOSPT	B	Not recommended	Not recommended	Yes
Intervention	Sensory Reeducation	INT_SR_raw	Exercise	-	-	Limited / Conflicting	Not addressed in CTS clinical practice guidelines	No
Intervention	Wrist orthosis (night)	INT_WONW_raw	Orthosis	JOSPT	B	Recommended	Recommended for night use	Yes
Intervention	Low-level laser (LLLT)	INT_LLLT_raw	Physical agents	JOSPT	B	Not recommended	Not recommended	Yes
Intervention	Extrinsic flexor strengthening	INT_EFMS_raw	Exercise	JOSPT	C	Limited / Conflicting	Limited evidence	No
Intervention	Analgesic electrical currents	INT_AEC_raw	Electrotherapy	JOSPT	C	Limited / Conflicting	May provide pain relief	No
Intervention	Intrinsic muscle strengthening	INT_IMS_raw	Exercise	JOSPT	C	Limited / Conflicting	Limited evidence	No
Intervention	Wrist orthosis (day+night)	INT_WO_raw	Orthosis	JOSPT	C	Limited / Conflicting	May be used if needed	No
Intervention	Iontophoresis	INT_Ionto_raw	Electrotherapy	JOSPT	B	Not recommended	Not recommended	Yes
Intervention	Neural mobilization	INT_NM_raw	Manual therapy	JOSPT	D	Limited / Conflicting	Conflicting evidence	No
Intervention	Functional electrical stimulation	INT_FES_raw	Electrotherapy	-	-	Limited / Conflicting	Not addressed in CTS clinical practice guidelines	No
Intervention	Wrist joint mobilization	INT_WJM_raw	Manual therapy	JOSPT	C	Limited / Conflicting	Limited evidence	No
Intervention	Superficial heating therapy	INT_SHT_raw	Physical agents	JOSPT	C	Limited / Conflicting	Short-term symptom relief	No
Intervention	Kinesio Taping	INT_KT_raw	Physical agents	AAOS	Limited	Limited / Conflicting	Not addressed in CTS clinical practice guidelines; insufficient evidence to support a formal recommendation	No
Intervention	Stretching (flex/ext)	INT_Strech_raw	Exercise	JOSPT	C	Limited / Conflicting	May be included	No
Intervention	Phonophoresis	INT_Phono_raw	Physical agents	JOSPT	C	Limited / Conflicting	May be used	No
Intervention	Referral OT/Psych	INT_OT_Psych_raw	Referral	-	-	Limited / Conflicting	Recommendation depends on the clinical context	No
Intervention	Short wave diathermy	INT_SWD_raw	Physical agents	JOSPT	C	Limited / Conflicting	May provide symptom relief	No
Intervention	Acupuncture / Dry needling	INT_AcupDNT_raw	Other	AAOS	Limited	Limited / Conflicting	Acupuncture was cited in the AAOS (2016) guideline as requiring further research, with no formal recommendation issued. Dry needling was not addressed in either guideline. Therefore, there is insufficient evidence to support a formal recommendation	No
Intervention	Cervical manual therapy	INT_C-SpineMT_raw	Manual therapy	JOSPT	C	Limited / Conflicting	May be considered	No

Abbreviations: AAOS, American Academy of Orthopaedic Surgeons; APL, abductor pollicis longus; APB, abductor pollicis brevis; BCTQ, Boston Carpal Tunnel Questionnaire; CTS, carpal tunnel syndrome; DASH, Disabilities of the Arm, Shoulder and Hand; JOSPT, Journal of Orthopaedic & Sports Physical Therapy; LLLT, low-level laser therapy; NRS, Numeric Rating Scale; OT, occupational therapy; Psych, psychology; SWMT, Semmes–Weinstein monofilament test; 2PD, two-point discrimination.
