# Supplementary material for: Clinical Decision‐Making and Guideline Alignment Among Brazilian Physical Therapists: Evidence From Standardized Carpal Tunnel Syndrome Vignettes
Source: J Eval Clin Pract. 2026 Aug 31;32(6):e70575. doi: 10.1111/jep.70575 (PMC13527589; doi:10.1111/jep.70575)
Supplement: Supplementary file 2 [file JEP-32-0-s002.docx]

# Supplementary Material S1

*The following standardized clinical vignettes were presented to all participants to assess clinical decision-making. For each vignette, participants selected the assessment and treatment strategies they considered appropriate using four response options (“Yes”, “Maybe”, “No”, and “I don’t know”).*

## Clinical vignette 1 – Assessment

A **47-year-old woman** with **overweight**, who works as a teacher, reports intermittent mild pain in both wrists and hands for approximately 12 months. Over the past 6 months, she has frequently awakened at night with numbness and tingling in her hands, predominantly affecting the palmar aspect of the thumb, index, and middle fingers. Her symptoms improve after shaking her hands or clenching her fist.

She reports no history of trauma, falls, or upper-limb surgery. Her diabetes mellitus and hypothyroidism are well controlled. She is physically inactive. Physical examination ruled out cervical radiculopathy, thoracic outlet syndrome, pronator teres syndrome, and compression neuropathies of the ulnar and radial nerves. There is no evidence of thenar muscle atrophy. She is not currently receiving corticosteroid therapy.

Based on the patient’s history and clinical examination, the most likely diagnosis is **mild carpal tunnel syndrome (CTS).**

## Clinical vignette 2 – Treatment

A **52-year-old woman** with **obesity**, who works as a hairdresser, reports pain in her right wrist and hand for more than two years. She complains of difficulty manipulating objects, reduced grip strength, and frequently dropping objects. She also reports difficulty fastening buttons.

At the time of assessment, her pain intensity is **6/10 on the Visual Analogue Scale (VAS)**. Physical examination reveals thenar eminence atrophy and reduced light-touch sensation, with sensation detected only using the **4.0-g red Semmes–Weinstein monofilament** over the volar aspect of the thumb, index, and middle fingers. Two provocative tests for carpal tunnel syndrome are positive.

There is no history of trauma, and plain radiographs of the wrist and hand show no abnormalities. Patient-reported outcome measures indicate moderate to severe difficulty performing hand-related activities (e.g., writing, bathing, dressing, carrying a shopping bag, and opening a jar), together with a **Boston Carpal Tunnel Questionnaire (BCTQ) Symptom Severity Scale score of 3.2**.

Cervical radiculopathy, double crush syndrome, and other compression neuropathies have been ruled out. Nerve conduction studies demonstrate **moderate CTS**, characterized by prolonged distal motor latency.
